# Fluid Shear Stress Increases Neutrophil Activation via Platelet-Activating Factor

**DOI:** 10.1016/j.bpj.2014.04.001

**Published:** 2014-05-20

**Authors:** Michael J. Mitchell, Kimberly S. Lin, Michael R. King

**Affiliations:** Department of Biomedical Engineering, Cornell University, Ithaca, New York

## Abstract

Leukocyte exposure to hemodynamic shear forces is critical for physiological functions including initial adhesion to the endothelium, the formation of pseudopods, and migration into tissues. G-protein coupled receptors on neutrophils, which bind to chemoattractants and play a role in neutrophil chemotaxis, have been implicated as fluid shear stress sensors that control neutrophil activation. Recently, exposure to physiological fluid shear stresses observed in the microvasculature was shown to reduce neutrophil activation in the presence of the chemoattractant formyl-methionyl-leucyl-phenylalanine. Here, however, human neutrophil preexposure to uniform shear stress (0.1–2.75 dyn/cm^2^) in a cone-and-plate viscometer for 1–120 min was shown to increase, rather than decrease, neutrophil activation in the presence of platelet activating factor (PAF). Fluid shear stress exposure increased PAF-induced neutrophil activation in terms of L-selectin shedding, *α*_M_*β*_2_ integrin activation, and morphological changes. Neutrophil activation via PAF was found to correlate with fluid shear stress exposure, as neutrophil activation increased in a shear stress magnitude- and time-dependent manner. These results indicate that fluid shear stress exposure increases neutrophil activation by PAF, and, taken together with previous observations, differentially controls how neutrophils respond to chemoattractants.

## Introduction

The initial recruitment and adhesion of leukocytes to the endothelial cell wall is a critical event in inflammation and lymphocyte homing to lymphatic tissues (1,2). In the initial phase of recruitment, free flowing leukocytes in postcapillary venules can be captured under the presence of hemodynamic shear forces as low as ∼0.4–0.5 dyn/cm^2^ (2,3), and subsequently exhibit rolling adhesion on the receptor-bearing endothelial cell wall. Rolling adhesion is mediated by rapid, force-dependent selectin-selectin ligand interactions between leukocytes and the endothelium, which can transition to firm adhesion and subsequent leukocyte transmigration into inflamed tissues (4,5). L-selectin, an important mediator on the leukocyte surface and constitutively expressed on the tips of microvilli, can initiate capture and rolling adhesion via binding to P-selectin glycoprotein ligand-1 on the endothelium (1,6,7). Mouse knockdown experiments using L-selectin-deficient mice show impaired neutrophil, lymphocyte, and monocyte migration into inflamed tissues (8), demonstrating the importance of L-selectin in inflammation and leukocyte homing. After initial tethering to the blood vessel wall, L-selectin can be rapidly cleaved from the cell surface by metalloprotease ADAM17, in a process known as shedding (9), to regulate cell rolling velocity and thus the rate of firm adhesion to the endothelium (10). In addition to L-selectin, *β*_2_ integrins on the surface of leukocytes bind with intercellular adhesion molecule-1 (ICAM-1) on the endothelium. Integrins such as macrophage-1 antigen (Mac-1), also known as CD11b/CD18 or integrin *α*_M_*β*_2_, and lymphocyte function-associated antigen-1 (LFA-1), also known as CD11a/CD18 or *α*_L_*β*_2_, are constitutively expressed on neutrophils and initially bind to ICAM-1 in an intermediate affinity state, which can result in slow rolling adhesion under low physiological shear forces (5). In response to increased signaling by chemotactic factors, the integrin molecules Mac-1 and LFA-1 undergo further conformational changes to achieve full activation, leading to firm adhesion. Together, the shedding of L-selectin and conformational activation of *β*_2_ integrins are key events that characterize neutrophil activation. One such chemotactic factor that induces neutrophil activation is platelet-activating factor (PAF) (11), which has been shown to decrease L-selectin expression and activation of *α*_M_*β*_2_ integrins on the neutrophil surface (12,13).

Hemodynamic shear forces have the ability to regulate the neutrophil activation response observed during inflammation. Human neutrophils exposed to static conditions (either in the presence or absence of chemoattractants) spread cytoplasm, project pseudopods, and migrate on glass substrates (14,15). Upon the application of fluid shear stress, neutrophils have been observed to retract pseudopods, assume a round resting state, and detach from glass substrates (14,15). Conversely, leukocytes exposed to centrifugation or treated with dexamethasone can reverse their response to shear stress, and project pseudopods upon fluid shear-stress exposure (16,17). Additionally, extended exposure to fluid shear stress can cause leukocyte membrane disruption (18).

G-protein coupled receptors (GPCRs) have been hypothesized to serve as molecular mechanosensors on the neutrophil surface. HL60 human leukemic cells differentiated into neutrophils using dimethylsulfoxide decreased GPCR constitutive activity in the presence of fluid shear stress, in addition to retraction of lamellipodia, and returned to a round resting state (19,20). Treatment with Gi inhibitor pertussis toxin or depletion of the GPCR formyl peptide receptor (FPR) via siRNA treatment significantly reduced shear-induced neutrophil pseudopod retraction (19). cDNA FPR transfection into undifferentiated HL60 cells with low FPR expression led to the projection of pseudopods, which quickly retracted in response to fluid shear stress exposure. Fluid shear stress preexposure has previously been shown to suppress neutrophil activation to formyl-methionyl-leucyl-phenylalanine (fMLP), which binds to FPR on the neutrophil surface during inflammation. fMLP is a chemotactic peptide derived from bacterial protein degradation and mitochondrial proteins upon tissue damage (21,22), and is present in low concentrations in the bloodstream during inflammation. Exposure to fluid shear stress in a cone-and-plate viscometer (0.1–4.0 dyn/cm^2^) before fMLP treatment significantly reduced fMLP-induced *α*_M_*β*_2_ integrin activation, L-selectin shedding, and pseudopod projection (23). Neutrophils increased their resistance to fMLP-induced activation in a fluid shear stress magnitude- and exposure time-dependent manner. Interestingly, fluid shear stress did not alter the neutrophil activation response to interleukin-8, which binds to GPCRs CXCR1 and CXCR2 on the neutrophil surface.

In addition to exposure to fMLP and interleukin-8 during inflammation, neutrophils can be exposed to platelet-activating factor (PAF), a phospholipid inflammatory mediator synthesized by monocytes, platelets, neutrophils, and endothelial cells (24). PAF can then activate platelets, neutrophils, and other leukocytes in the bloodstream, particularly during inflammation and allergy (25). Recognition by these cells occurs via the PAF receptor (PAFR), a GPCR on the neutrophil surface, which leads to neutrophil migration through intercellular junctions in the endothelium to ameliorate tissue injury (26,27). After synthesis via endothelial cells, PAF is translocated to the cell surface rather than secreted into the bloodstream, where it is available for binding to blood cells (25). This is in contrast to fMLP, which is released from tissues as the cleavage products of bacterial and mitochondrial proteins. Upon recognition via interaction with PAF receptors on the surface of neutrophils, PAF induces cell adhesion and cell polarization, enhanced motility, priming of granular enzyme release, and redistribution of surface adhesion ligands in neutrophils (13). These physiological effects occur while PAF remains associated with the endothelial membrane, showing that PAF commonly acts by juxtacrine signaling, which is necessary for tightly localized neutrophil recruitment (28). Additionally, differences in functional response have been observed with PAF and fMLP. For example, neutrophils stimulated by fMLP can produce superoxide ions, whereas minimal ions are produced in neutrophils stimulated by PAF (29). The different physiological responses to inflammation suggest that different intracellular pathways are utilized by PAFR and FPR, and that the response depends on the type of chemoattractant present (i.e., PAF or fMLP).

The effect of hemodynamic shear forces on early indicators of PAF-induced neutrophil activation remains unknown. In this study, we quantified the effects of fluid shear stress pretreatment on PAF-induced neutrophil activation.

## Materials and Methods

### Reagents

APC-conjugated mouse anti-human CD62L specific for human L-selectin, APC-conjugated mouse IgG_1_ isotype control antibody, and fluorescein isothiocyanate (FITC)-conjugated mouse IgG_1_ isotype control antibody were purchased from BD Biosciences (San Jose, CA). FITC-conjugated mouse anti-human CBRM1/5, which binds to the activation epitope of CD11b, was purchased from eBioscience (San Diego, CA). Platelet-activating factor (PAF) was purchased from Millipore (Billerica, MA). Primary goat anti-human PAF-R antibody, which binds to the extracellular N-terminus of the PAF receptor, and FITC-conjugated secondary donkey anti-goat IgG antibody were purchased from Santa Cruz Biotechnology (Santa Cruz, CA). Tumor necrosis factor-*α* protease inhibitor-0 (TAPI-0) and p38 mitogen-activated protein (MAP) kinase inhibitor SB203580 were purchased from Peptides International (Louisville, Kentucky) and Millipore, respectively. Human TruStain FcX Fc receptor blocking solution was purchased from Biolegend (San Diego, CA). Ca^2+^ and Mg^2+^ free Hank’s balanced salt solution (HBSS) and Dulbecco’s phosphate-buffered saline (DPBS) were purchased from Invitrogen (Carlsbad, CA). Endotoxin-free water was purchased from MO Bio (Carlsbad, CA). Endotoxin-free human serum albumin HEPES, low-endotoxin, and essentially globulin-free bovine serum albumin were purchased from Sigma Aldrich (St. Louis, MO).

### Neutrophil isolation

Primary human neutrophils were isolated as described previously in Mitchell et al. (30,31). Whole peripheral blood was obtained via venous needle injection from healthy human donors after informed consent. Neutrophils were separated by centrifugation at 480 × *g* at 23°C for 50 min in a Marathon 8 K centrifuge (Fisher Scientific, Pittsburgh, PA) using 1-Step Polymorphs (Accurate Chemical and Scientific Corporation, Westbury, NY), and resuspended in Mg^2+^- and Ca^2+^-free HBSS to remove excess polymorph solution. Remaining red blood cells were lysed hypotonically, and purified neutrophils were resuspended in Mg^2+^-free HBSS buffer with 0.5% human serum albumin, 10 mM HEPES, and 2 mM Ca^2+^ at a pH of 7.4 at a concentration of 0.5 × 10^6^ cells/mL. Isolation protocols were approved by the Institutional Review Board of Cornell University.

### Cone-and-plate viscometer assay

Cells were exposed to uniform fluid shear stress in a cone-and-plate DV-II+ Pro Digital Viscometer (Brookfield Engineering Laboratories, Middleboro, MA) as previously described in Mitchell and King (23,32) and Mitchell et al. (33). Neutrophils were placed in a plate underneath a cone angled at 0.8°. Shear rate, *G*, is known to be a function of cone angle and independent of distance from the center by

(1) $$G=\frac{\omega }{\tan\;\theta },$$

where *ω* is the angular velocity of the cone (rad/s) and *θ* is the cone angle (rad) (23). To achieve a desired shear stress, *τ*, assuming Newtonian fluid behavior, the shear rate was varied according to

(2) τ=μG,

where *μ* is the viscosity of the buffer solution (Pa·s) (32). Before fluid shear-stress exposure, the cone and plate were incubated with 5% bovine serum albumin for 1 h to prevent nonspecific adherence of neutrophils to the steel surfaces. Neutrophil suspensions were then placed onto the plate at a concentration of 0.5 × 10^6^ cells/mL. Neutrophils were exposed to fluid shear stress (0.1–2.75 dyn/cm^2^) for 1–120 min at 23°C. Cells were then immediately incubated with 1 *μ*M PAF for 10 min at room temperature. Cells were either washed and prepared for antibody labeling or fixed in paraformaldehyde and prepared for morphology analysis. For L-selectin inhibition studies, neutrophils were treated with 25 *μ*M TAPI-0, an inhibitor of the L-selectin sheddase ADAM-17, or 1 *μ*M SB203580, an inhibitor of p38 MAP kinase, for 60 min before fluid shear stress exposure. Neutrophil viability levels of >95%, before and after cone-and-plate assays, were confirmed using a Trypan Blue exclusion dye.

### Flow cytometry

Untreated and PAF-treated neutrophils were incubated with APC-conjugated mouse anti-human CD62L and FITC-conjugated mouse anti-human CBRM1/5 antibodies after PAF treatment. Control samples to measure nonspecific binding were incubated with APC-conjugated and FITC-conjugated mouse IgG_1_ isotype control antibodies. All samples were incubated at 4°C for 35 min and washed twice with cold Ca^2+^-free and Mg^2+^-free DPBS. Data were collected and analyzed using an Accuri C6 Flow Cytometer (BD Biosciences, San Jose, CA) and FCS Express V3 (De Novo Software, Thornville, Ontario, Canada) software.

### Bright-field microscopy and imaging analysis

Neutrophils were prepared for morphology analysis via fixation with 4% paraformaldehyde (Electron Microscopy Sciences, Hatfield, PA) for 30 min at 4°C. Cells were washed and placed in a 24-well plate, and imaged via bright-field and phase contrast microscopy using a model No. IX81 Inverted System Microscope (Olympus America, Center Valley, PA). The software METAMORPH (Molecular Devices, Sunnyvale, CA) was used to threshold outlines of neutrophils. Neutrophil circularity was quantified by calculating the shape factor, defined by

(3) $$\text{Shape}\;\text{factor}=\frac{4\pi A}{P^{2}},$$

where *A* = area (*μ*m^2^) and *P* = perimeter (*μ*m) (23). A shape factor of 1 defines a perfect circle. All shape factor data were imported into the software EXCEL (Microsoft, Redmond, WA) for analysis.

### PAF receptor expression

Neutrophil surface expression of PAF receptor (PAFR) was detected using flow cytometry. Cells were incubated in Human TruStain FcX for 10 min at room temperature, to block nonspecific binding between Fc regions of antibodies and Fc receptors on the cell surface. Neutrophils were then labeled with PAFR antibody (N-17) and incubated for 1 h at 4°C. After washing twice with cold Ca^2+^ free and Mg^2+^ free DPBS, samples were incubated with FITC-conjugated secondary IgG antibody for 30 min at 4°C. Samples were washed twice and analyzed using a flow cytometer. To measure PAF receptor density of neutrophils exposed to static conditions and fluid shear stress, PAF receptor fluorescence intensity measurements were compared to the fluorescence of standard microspheres with known levels of fluorescence. Quantum FITC-5 molecules of equivalent soluble fluorochrome kits (MESF; Bangs Laboratories, Fishers, IN), consisting of four types of microspheres with increasing levels of FITC fluorochrome and one blank microsphere population, were used to create a fluorescence calibration curve according to the manufacturer’s instructions. Quantum FITC-5 MESF beads were analyzed for fluorescence intensity using a flow cytometer to create a standard fluorescence curve. Neutrophils exposed to static and sheared conditions, which were labeled with PAF receptor and FITC-conjugated secondary antibodies, were then analyzed for fluorescence intensity using a flow cytometer. PAF receptor density was estimated using the standard fluorescence curve.

### Statistical analysis

Shape factor and flow cytometry data were plotted using the software GRAPHPAD PRISM 5 (GraphPad Software, La Jolla, CA). Statistical two-tailed paired *t*-tests were performed to test for significant differences between data sets, with *p* < 0.05 being considered significant.

## Results

### Fluid shear stress increases neutrophil PAF-induced L-selectin shedding and *α*_M_*β*_2_ integrin activation

**Figure 1:**
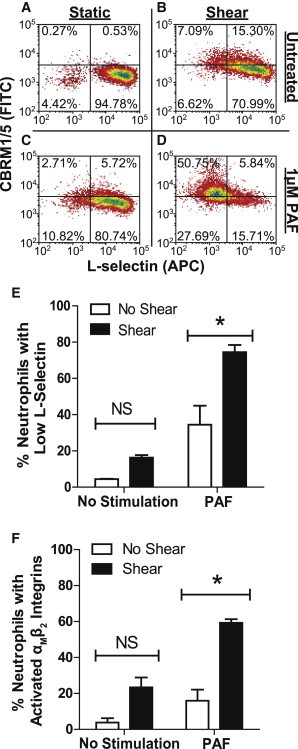
Fluid shear stress exposure and PAF increase neutrophil activation. Neutrophils exposed to (*A*) static and (*B*) shear conditions at 1.0 dyn/cm^2^ for 2 h. PAF-treated samples were exposed to 1 *μ*M PAF for 10 min (*C* and *D*) and analyzed for activation. Upper two quadrants signify activated *α*_M_*β*_2_ integrin subunit expression. Two right hand quadrants show L-selectin expression. Gating was determined using fluorescence intensities of isotype controls. Bar graph representation of (*E*) L-selectin shedding and (*F*) *α*_M_*β*_2_ integrin activation. Low L-selectin denotes neutrophils that have shed L-selectin from their surface, and have fluorescence intensities no greater than that of isotype controls. Conditions were repeated for three donors (*n* = 3). Error bars signify 95% confidence intervals. ^∗^*P* < 0.05. *NS*, not significant. To see this figure in color, go online.

We initially examined the response of neutrophils to PAF under fluid shear stress conditions in terms of L-selectin shedding and *α*_M_*β*_2_ integrin activation using a cone-and-plate viscometer, which has been previously used by our lab to examine fluid shear stress effects on neutrophils and cancer cells (23,32,34). Neutrophils were in static conditions or exposed to fluid shear stress of 1.0 dyn/cm^2^ for 2 h and subsequently treated with 1 *μ*M PAF for 10 min at 23°C. PAF, a phospholipid produced by endothelial cells that acts as a mediator of the inflammatory response (13), is known to increase L-selectin shedding and induce structural change in *α*_M_*β*_2_ integrins to an activated conformation (35). Negligible neutrophil L-selectin shedding and *α*_M_*β*_2_ integrin activation was observed in samples exposed to static conditions (Fig. 1 *A*), whereas fluid shear stress exposure induced a moderate increase in both indicators of activation (Fig. 1 *B*). However, in response to PAF, neutrophils showed a greater degree of L-selectin shedding and *α*_M_*β*_2_ integrin activation after fluid shear stress preexposure (Fig. 1 *D*), compared to preexposure to static conditions (Fig. 1 *C*). Although trends indicate that shear stress exposure alone does increase neutrophil L-selectin shedding and *α*_M_*β*_2_ integrin activation, these changes were found to be not significant (Fig. 1, *E* and *F*). In the presence of PAF, the average percentage of neutrophils that shed L-selectin increased significantly after shear stress exposure, from ∼35% for cells under static conditions to >70% for cells exposed to fluid shear stress (Fig. 1 *E*). Similarly, the average percentage of neutrophils expressing the activated *α*_M_*β*_2_ integrin subunit increased in PAF-treated samples, from ∼15% for cells under static conditions to ∼60% for cells exposed to fluid shear stress (Fig. 1 *F*). These results suggest that fluid shear stress preexposure and PAF can both act to increase neutrophil activation.

### PAF activation of neutrophils increases with fluid shear stress magnitude

**Figure 2:**
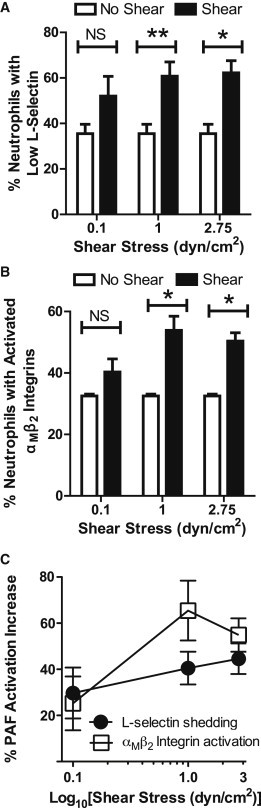
PAF activation of neutrophils is fluid shear stress magnitude-dependent. Cells were exposed to a range of shear stress (0.1–2.75 dyn/cm^2^) for 30 min at 23°C, followed by immediate treatment with 1 *μ*M PAF for 10 min. Quantification of (*A*) L-selectin shedding and (*B*) activated *α*_M_*β*_2_ integrin expression on neutrophils after exposure to increasing shear stress. (*C*) PAF activation increase, in terms of L-selectin shedding and *α*_M_*β*_2_ integrin activation, plotted as a function of the log_10_ of shear stress. ^∗^*P* < 0.05, ^∗∗^*P* < 0.01.

To assess the effect of fluid shear stress magnitude on neutrophil PAF-induced activation, cells were exposed to shear stresses ranging from 0.1 to 2.75 dyn/cm^2^ in a cone-and-plate viscometer for 30 min, followed by treatment with 1 *μ*M PAF for 10 min at 23°C. This range of shear stress includes that typically found in the venular microcirculation (36), which is the primary site of leukocyte interactions with the endothelium (37,38). No differences in L-selectin shedding or *α*_M_*β*_2_ integrin activation were seen after exposure to low fluid shear stress of 0.1 dyn/cm^2^ (Fig. 2, *A* and *B*). However, a significant increase in neutrophil PAF-induced L-selectin shedding was observed after exposure to shear stresses of 1.0 and 2.75 dyn/cm^2^, compared to static conditions (Fig. 2 *A*). Fluid shear stress induced a similar increase in activation in terms of the activated *α*_M_*β*_2_ integrin epitope, when compared to neutrophils stimulated with PAF under static conditions (Fig. 2 *B*).

To identify the increase in shedding of L-selectin and activated *α*_M_*β*_2_ integrin subunit expression with respect to fluid shear stress, the neutrophil sensitization responses to L-selectin shedding and *α*_M_*β*_2_ integrin activation were calculated using the following equation:

(4) $$\%\;\text{PAF}\;\text{activation}\;\text{increase}=\frac{\left(\%\;{\text{Cells}}_{\text{Shear}}\right)-\left(\%\;{\text{Cells}}_{\text{Static}}\right)}{\%\;{\text{Cells}}_{\text{Static}}}\times 100\%.$$

When shear-stress magnitude was varied, it was found that the PAF activation increase in neutrophils increased with increasing magnitude of fluid shear stress preexposure (Fig. 1 *C*).

### Neutrophil PAF activation is dependent on fluid shear stress exposure time

**Figure 3:**
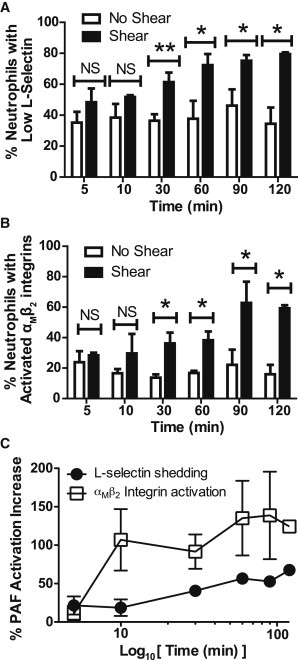
Increased neutrophil PAF activation is fluid shear stress time-dependent. Cells were sheared (1.0 dyn/cm^2^) for a duration of 5–120 min at 23°C and immediately incubated in 1 *μ*M PAF for 10 min. Quantification of (*A*) L-selectin shedding and (*B*) activated *α*_M_*β*_2_ integrin expression of neutrophils over time. (*C*) Percent PAF activation increase, in terms of L-selectin shedding and *α*_M_*β*_2_ integrin activation, plotted as a function of the log_10_ of time. ^∗^*P* < 0.05, ^∗∗^*P* < 0.01.

To study the kinetics of the neutrophil PAF activation in the presence of fluid shear stress, neutrophils were sheared at 1.0 dyn/cm^2^ for durations ranging from 5 to 120 min at 23°C, followed by stimulation with 1 *μ*M PAF. Whereas no significant differences in L-selectin shedding were observed after 10 min shear stress exposure, L-selectin shedding significantly increased after shear stress exposure for 30 min (Fig. 3 *A*). A significant increase in PAF-induced *α*_M_*β*_2_ integrin activation was also observed after 30 min of exposure to shear stress conditions (Fig. 3 *B*). The percent PAF activation increase equation used to calculate the shear stress dose-response plot was also used to quantify the increase in neutrophil PAF activation over shear stress exposure time. With increasing durations of shear stress preexposure, neutrophils stimulated with PAF increased their level of activation (Fig. 3 *C*). By 30 min, the percent increase in L-selectin shedding and *α*_M_*β*_2_ integrin activation reached ∼95% and 45%, respectively.

### Fluid shear stress increases PAF-induced neutrophil morphological changes

**Figure 4:**
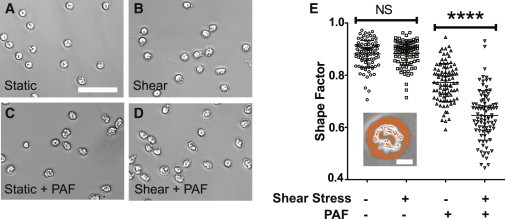
Fluid shear stress increases neutrophil morphological changes in the presence of PAF. Representative bright-field images of untreated neutrophils after exposure to (*A*) static and (*B*) shear conditions (1.0 dyn/cm^2^) for 30 min, compared to cells stimulated with 1 *μ*M PAF after exposure to (*C*) static and (*D*) shear conditions. Scale bar = 50 *μ*m. (*E*) Shape factor data of neutrophils exposed to static (1.0 dyn/cm^2^) or shear conditions, with and without 1 *μ*M PAF stimulation. (*Inset*) Images were thresholded to calculate shape factor of cells (scale bar = 5 *μ*m). *n* = 3 donors, with >100 neutrophils from each donor analyzed for shape factor. To see this figure in color, go online.

In addition to L-selectin shedding and *α*_M_*β*_2_ integrin activation, cell polarization is among the responses exhibited in activated neutrophils after PAF stimulation (13). During this phenomenon, neutrophils extend pseudopods to enable cell motility, allowing neutrophils to effectively extravasate through the blood vessel wall and migrate to inflamed tissue during the immune response (39). To assess the effect of fluid shear stress on cell polarization, neutrophils were exposed to 1.0 dyn/cm^2^ shear stress for 30 min, followed by stimulation with PAF. Cells were then fixed with 4% paraformaldehyde and examined for morphological changes. Qualitative evaluation of neutrophil morphology in the absence of PAF under either static conditions or 1.0 dyn/cm^2^ fluid shear stress showed similar round morphologies (Fig. 4, *A* and *B*) characteristic of resting neutrophils. Upon stimulation with PAF, neutrophils assumed a notably more elongated form with extended pseudopods (Fig. 4 *C*), whereas cells exposed to shear before PAF stimulation displayed more exaggerated features than those under static conditions (Fig. 4 *D*). To calculate neutrophil shape factor, images were thresholded to select the outline of the cells to be measured (Fig. 4 *E*). Neutrophils exposed to static and shear conditions in the absence of PAF stimulation both exhibited shape factors close to 1 and were not significantly different from each other (Fig. 4 *F*), consistent with empirical observations. Although neutrophils stimulated with PAF after exposure to static and shear conditions displayed shape factor values significantly less than those in the absence of PAF, PAF-treated neutrophils exposed to shear stress showed significantly lower average shape factor than those exposed to static conditions (Fig. 4 *F*). These results indicate that fluid shear stress enhances changes in neutrophil morphology in response to PAF.

### Neutrophil PAF surface receptor expression is unaltered in response to fluid shear stress

**Figure 5:**
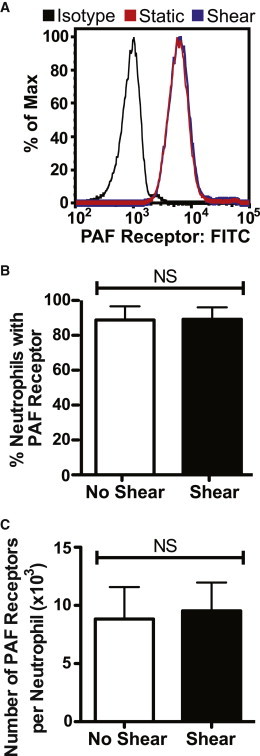
Fluid shear stress does not alter PAF receptor expression. Neutrophils were exposed to fluid shear stress (1.0 dyn/cm^2^) for 30 min at 23°C. Flow cytometry histograms (*A*) and mean fluorescence intensities (*B*) of PAF receptor expression in sheared and nonsheared neutrophil samples. (*C*) PAF receptor density of neutrophils exposed to static conditions and fluid shear stress (1.0 dyn/cm^2^) for 30 min at 23°C. *NS*, not significant. To see this figure in color, go online.

Previous studies showed that neutrophil response to fluid shear stress can result in changes in chemoattractant surface receptor expression (23). To investigate whether neutrophil response to fluid shear stress alters the expression of the PAFR on the cell surface, neutrophils were exposed to fluid shear stress (1.0 dyn/cm^2^) for 30 min, labeled with fluorescent anti-PAFR antibodies, and analyzed via flow cytometry. The distribution of PAFR surface expression among the neutrophil population remained unchanged between static and shear conditions (Fig. 5 *A*). PAFR surface expression was quantified in terms of percent of neutrophils expressing PAF receptor, and results show no significant change in expression between neutrophils under static and shear conditions (Fig. 5 *B*). To measure PAF receptor densities of neutrophils exposed to shear and static conditions, quantum MESF beads were used to generate a fluorescence standard curve to estimate the number of PAF receptors per neutrophil. No significant differences in the number of PAF receptors per neutrophil were found, as neutrophils exposed to either shear or static conditions averaged ∼9000 PAF receptors/cell (Fig. 5 *C*).

### Shear stress and PAF-induced neutrophil L-selectin shedding is ADAM 17- and p38 MAP kinase-dependent

**Figure 6:**
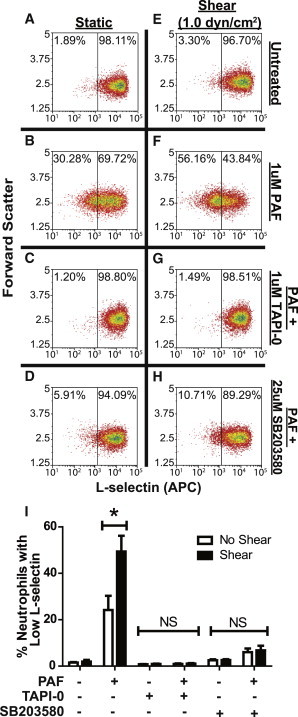
Shear- and PAF-induced L-selectin shedding is ADAM 17- and p38 MAP kinase-dependent. L-selectin expression as a function of forward scatter of nonsheared and sheared (1.0 dyn/cm^2^) neutrophils in the absence of PAF (*A* and *B*) after treatment with PAF (*C* and *D*), and PAF-stimulated cells treated with TAPI-0 (*E* and *F*) and SB203580 (*G* and *H*). Gate determined using fluorescence of isotype controls. (*I*) Quantification of L-selectin shedding in all samples. *n* = 3 separate donors. *^∗^P <* 0.05. *NS*, not significant. To see this figure in color, go online.

Neutrophil shedding of L-selectin is dependent on the p38 mitogen-activated protein kinase pathway (40). This pathway subsequently leads to activation of ADAM-17, a protease involved in TNF-*α* activation and known to regulate L-selectin shedding (41). To shed light upon the mechanism by which L-selectin is shed in the presence of shear stress preexposure followed by PAF stimulation, neutrophils were treated with 25 *μ*M TAPI-0 (Fig. 6 *C*) or 1 *μ*M SB203580 (Fig. 6 *D*), inhibitors of p38 MAP kinase and ADAM-17 sheddase, respectively, for 60 min before shear stress exposure. Untreated and PAF-treated neutrophils maintained in static conditions (Fig. 6, *A*, *C*, *E*, and *G*) or fluid shear stress (Fig. 6, *B*, *D*, *F*, and *H*) were then labeled with fluorescent anti-L-selectin antibodies. Expression of L-selectin on inhibitor-treated cells was then compared to uninhibited cells with and without PAF treatment. Consistent with previous work, static neutrophils treated with TAPI-0 or SB203580 and subsequently stimulated with PAF showed minimal L-selectin shedding, relative to untreated neutrophils (Fig. 6 *I*). After shear stress exposure, sensitization to PAF-induced L-selectin shedding was attenuated in TAPI-0 and SB203580-treated neutrophils (Fig. 6, *E*–*I*). These results suggest that fluid shear stress- and PAF-induced L-selectin shedding is ADAM 17- and p38 MAP kinase-dependent.

## Discussion

The goal of this study was to characterize the effect of fluid shear stress on PAF-induced L-selectin shedding, *α*_M_*β*_2_ integrin activation, and morphological changes in neutrophils. Our results show that neutrophil PAF activation is significantly increased after fluid shear stress preexposure of magnitude as low as 1.0 dyn/cm^2^, consistent with previous studies. Marschel and Schmid-Schönbein (42) observed rapid pseudopod retraction of neutrophils adhering via *β*_2_ integrins on a glass substrate upon fluid shear stress exposure at 1.0 dyn/cm^2^. In terms of initial rolling adhesion, Finger et al. (3) reported that a minimal wall shear stress of 0.4 dyn/cm^2^ is required for stable tethering and rolling of neutrophils via L-selectin on peripheral lymph node addressin, with a maximal number of neutrophils rolling at 1.0 dyn/cm^2^. Sundd et al. (2) reported that fluid shear stresses >0.5 dyn/cm^2^ are required for neutrophil rolling on P-selectin. Such adhesion is a necessary precursor interaction to binding with PAF on endothelial cells during the onset of inflammation.

**Figure 7:**
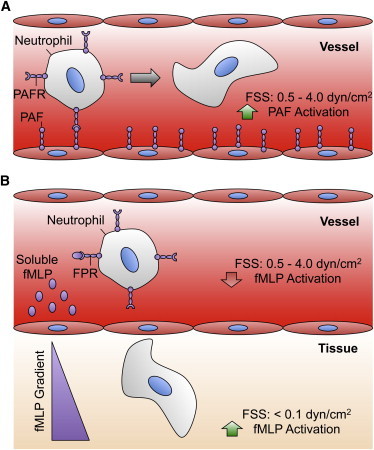
Comparison of fluid shear stress effects on neutrophil activation via PAF (*A*) and fMLP (*B*). Shear stress exposure in the microvasculature increases neutrophil activation via PAF (*A*), whereas activation is suppressed upon exposure to fMLP (*B*). (*FPR*, formyl peptide receptor; *FSS*, fluid shear stress; *PAF*, platelet-activating factor; *PAFR*, platelet-activating factor receptor.). To see this figure in color, go online.

Interestingly, our previous study characterizing the effect of fluid shear stress on fMLP-induced L-selectin shedding and *α*_M_*β*_2_ integrin activation in neutrophils showed that, in stark contrast to the findings in this article, neutrophils display a resistance instead of a sensitization to chemoattractant stimulation (23). fMLP is a peptide chemoattractant released by certain bacteria and mitochondria at a site of tissue infection and/or injury, and activates neutrophils by interacting with the formyl peptide receptor on the neutrophil surface (Fig. 7) (43). In humans, fMLP is present only at very low concentrations in the bloodstream, compared to sites of tissue infection and/or injury. Sustained activation to fMLP stimuli in the bloodstream could hinder the neutrophil response to infection by promoting pseudopod projection, increasing neutrophil transit time in blood, and enhancing neutrophil retention in the microvasculature (44,45). Thus, if neutrophils preexposed to hemodynamic shear forces in the bloodstream increased fMLP-induced activation, neutrophils would be fully activated in the bloodstream and less efficient at transmigrating into localized tissues. Additionally, activation of neutrophils via fMLP also induces production of reactive oxygen species (46), and thus shear-induced resistance to fMLP can reduce reactive oxygen species-induced damage to healthy blood vessels. By developing a resistance to fMLP-induced activation when exposed to shear in the bloodstream, neutrophils avoid unnecessary activation in the bloodstream, facilitating extravasation and subsequent migration in tissues. In contrast, PAF-induced activation of neutrophils in the bloodstream is physiologically important (Fig. 7).

PAF is a unique chemotactic factor in that it triggers both thrombotic and acute inflammatory responses after stimulation by chemical mediators found in the blood, such as thrombin and histamines, and thus links the hemostatic and innate immune responses (47). For platelets, exposure to PAF is important for activation and aggregation at sites of vascular injury. For neutrophils, it primes the release of granular factors, which play a role in coagulation (13). Because it is secreted by various leukocytes in the fluid phase, PAF is thought to have endocrine and paracrine signaling roles as well, which may also necessitate its presence in the vascular system for its use as a means of transport (13). The increase in neutrophil PAF activation in the presence of fluid shear stress primes neutrophils for activation in the bloodstream, allowing them to respond to vascular injury (47). The differences in PAF and fMLP-induced activation after shear stress pretreatment suggest that there is an underlying mechanism for neutrophils to optimize their response to their local fluid shear stress microenvironment (Fig. 7).

Although the observed sensitization response was not coupled with a shear stress-induced change in PAF receptor density on the neutrophil surface, sensitization to L-selectin shedding was shown to be p38 MAP kinase (MAPK)-dependent. The molecular mechanisms underlying *α*_M_*β*_2_ integrin activation were not probed, as they remain poorly understood and are the subject of ongoing research (48,49). Although this study focused on mechanical force-induced increases to PAF-induced activation, increases in neutrophil PAF activation have previously been induced by chemical exposure. Human recombinant granulocyte macrophage colony stimulating factor (GM-CSF) was shown to prime arachidonic acid release and intracellular calcium fluxes in neutrophils after PAF stimulation (50). One proposed hypothesis for this phenomenon is that GM-CSF remodels the lipid profile of the neutrophil plasma membrane, causing receptors on the neutrophil surface to become more accessible to substrates (50). This hypothesis may also explain the observed increases in PAF activation; shear forces may act on the neutrophil membrane to readily expose the PAFR for easier access by immobilized PAF, without changing receptor density. Ginkgolide B, a bioactive component of Ginkgo biloba leaf extract 761, is another chemical found to prime neutrophils for activation. Ginkgolide B primes fMLP- and zymosan-induced respiratory burst in neutrophils by acting through the PAF receptor. Although this mechanism remains unknown, it is hypothesized that this priming effect may involve phosphorylation or some conformational change of PAFR to a more activated form. It has been suggested by a number of studies in response to observations of heterogeneous low- and high-affinity binding of PAFR to PAF that PAFR may exist in various conformational states and requires activation to achieve high-affinity binding (51,52). Mutations of the Ala^230^ and Leu^231^ residues on the third intracellular loop of the receptor to Glu^230^ and Arg^231^, respectively, have been shown to lead to inactive and constitutively active PAFR states (53). This finding suggests the possibility that fluid shear stress may affect the PAF receptor directly, and future work could probe conformational changes or clustering of the receptor in response to shear.

In response to PAF stimulation, PAFR, much like other GPCRs, can internalize into cells with their respective PAF ligands to cause either desensitization or resensitization of the receptor (54–56). Immediately after PAF binding, PAFR uncouples from G-proteins, and is rapidly phosphorylated by different kinases. During desensitization, PAFR can undergo endocytosis into endosomes and be targeted for degradation via ubiquitination (55). This can lead to an overall downregulation of PAFR, and thus desensitization in the presence of PAF ligands. Conversely, resensitization to PAF can occur, in a process where PAFR is thought to move into early endosomes and recycle to the cell membrane. Previous work by our group showed that FPR can internalize within neutrophils upon exposure to fluid shear stress, in the absence of fMLP stimulation (23). In this study, there were no differences found in PAFR expression on the neutrophil surface after fluid shear stress exposure, thus suggesting that fluid shear stress preexposure does not desensitize neutrophils to PAF stimulation. However, it is possible that fluid shear stress preexposure could affect PAFR internalization in the presence of the agonist, perhaps by enhancing recycling of PAFR, which would explain the increase in neutrophil PAF activation.

Neutrophil p38 MAPK signaling may also be influenced by fluid shear stress exposure. Killock and Ivetić (57) showed that stimulation of resting leukocytes with calcyculin A or cantharidin led to increased levels of phosphorylated p38 MAPK, which triggers increased phosphorylation of the cytoplasmic tails of TNF-*α*-converting enzyme and its subsequent expression on the cell surface, which likely caused the simultaneous increase of L-selectin shedding. Fluid shear stress may increase phosphorylation of p38 MAPK in a similar manner, to induce the sensitized L-selectin shedding response observed in our study. To explore this possible mechanism, future efforts could compare the surface expression of TNF-*α*-converting enzyme or radioactively-labeled phosphorylated p38 MAPK pre- and post-shear stress exposure. Downstream of PAFR signaling, PAF, like fMLP, is known to strongly activate MAP kinase kinase-3. Unlike fMLP, PAF minimally activates MAP extracellular signal-regulated kinase (ERK) kinase kinase-1, Raf, and the p42/44 (ERK) MAP kinases, all of which are pathways that are strongly activated by fMLP (29). Fluid shear stress could act to increase activation of these pathways to enhance neutrophil PAF-induced activation.

One important difference between the experimental conditions of this study and the physiological conditions of the human body is the presentation of PAF. Physiologically, whereas PAF is secreted by many leukocytes in a fluid-phase form, in the inflammatory response it is most commonly synthesized by endothelial cells and is translocated onto the cell surface, where it primarily acts through a juxtacrine signaling mechanism. The main advantage to the cell-associated form of communication is to allow for spatial regulation of signaling and only permit localized activation, thus preventing activation of PMNs in free-flowing blood (58). Because unregulated secretion of PAF has been shown to induce anaphylactic or septic shock and trauma (47), the body has adopted various mechanisms to regulate plasma PAF concentrations. One method of control is by PAF acetylhydrolase, which degrades PAF and limits its half-life to no more than a few minutes in the blood stream (13). The half-life of PAF in a study involving patients with acute allergic reactions was found to be ∼13.6 min in serum with the lowest PAF acetylhydrolase activity, ∼6.0 min in serum with intermediate PAF acetylhydrolase activity, and ∼3.8 min in serum with the highest PAF acetylhydrolase activity (59).

Our experiments were conducted in the absence of PAF acetylhydrolase, and neutrophils were treated with PAF for a duration of 10 min. Thus, we expect there to be less neutrophil activation than that reported in our results in individuals with intermediate to high PAF acetylhydrolase activity. In addition to limiting the actions of PAF in the bloodstream, the body also has mechanisms to facilitate neutrophil recognition of PAF. One example of this involves granule membrane protein-140, a membrane glycoprotein in the granules of platelets and endothelial cells that is translocated to the cell surface along with PAF upon stimulation by histamine or thrombin. It is found to act cooperatively with PAF in facilitating recruitment of *β*_2_ integrins on neutrophils in an indirect manner—it initiates tethering of neutrophils to endothelial cells, bringing the otherwise inaccessible and immobilized PAF in proximity to its receptor on neutrophils (60). In comparison, the fluid phase form of the chemoattractant in our study may make PAF more accessible to neutrophils and heighten the response. Although the discrepancy between the experimental conditions of our study and those of in vivo conditions may cause differences in the degree of neutrophil activation, our reported findings still hold—fluid shear stress sensitizes PAF-induced L-selectin shedding, *α*_M_*β*_2_ integrin activation, and morphological changes of neutrophils during the inflammatory response. Future work that explores the role of other enzymes in the p38 MAPK pathway downstream of PAFR, potential cross-talk mechanisms between integrins, and the possibility of different conformational states in the receptor itself may bring a greater understanding of the molecular details of the role of fluid shear stress in the innate immune response.
